# Comparative mitochondrial genomics toward exploring molecular markers in the medicinal fungus *Cordyceps militaris*

**DOI:** 10.1038/srep40219

**Published:** 2017-01-10

**Authors:** Shu Zhang, Ai-Jing Hao, Yu-Xiang Zhao, Xiao-Yu Zhang, Yong-Jie Zhang

**Affiliations:** 1 Institute of Applied Chemistry, Shanxi University, Taiyuan 030006, China; 2 School of Life Sciences, Shanxi University, Taiyuan, Shanxi 030006, China; 3 Farm Product Storage and Freshening Institute, Shanxi Academy of Agricultural Sciences, Taiyuan, Shanxi 030031, China

## Abstract

*Cordyceps militaris* is a fungus used for developing health food, but knowledge about its intraspecific differentiation is limited due to lack of efficient markers. Herein, we assembled the mitochondrial genomes of eight *C. militaris* strains and performed a comparative mitochondrial genomic analysis together with three previously reported mitochondrial genomes of the fungus. Sizes of the 11 mitochondrial genomes varied from 26.5 to 33.9 kb mainly due to variable intron contents (from two to eight introns per strain). Nucleotide variability varied according to different regions with non-coding regions showing higher variation frequency than coding regions. Recombination events were identified between some locus pairs but seemed not to contribute greatly to genetic variations of the fungus. Based on nucleotide diversity fluctuations across the alignment of all mitochondrial genomes, molecular markers with the potential to be used for future typing studies were determined.

*Cordyceps militaris*, which generally parasitizes larva or pupa of lepidopteran insects, is the type species of the genus *Cordyceps* (Hypocreales, Ascomycetes). This fungus is distributed worldwide from 0 to >2000 m above sea level^1^. Its fruiting body has now been mass-produced artificially and developed into health food (i.e. not just nutritious in an ordinary sense, but eaten specifically for its health-promoting properties), and the species is one of the most representative and widely-used species in *Cordyceps* sensu lato^1^. Biologically active compounds (e.g., cordycepin, polysaccharides, cordymin) isolated from the fungus exhibit a variety of pharmacological effects, including anti-cancer, antioxidant, anti-inflammatory, immune-enhancing, or antifungal activities^2^,^3^,^4^,^5^. In addition to the interest in artificial cultivation and pharmacological effects, researchers have studied the fungus broadly from the viewpoints of genomics^6^, transcriptomics^7^,^8^, methylome^9^, and proteomics^7^.

As a species with a worldwide distribution and a broad host range^1^, *C. militaris* serves as an ideal material to understand evolutionary biology of fungi. Our knowledge about the intraspecific genetic diversity of *C. militaris*, however, has been rather limited, and its population genetic structure is far from clear due to lack of highly efficient molecular markers. For example, the maximum kimura-2-parameter genetic distance based on nrDNA ITS sequences among *C. militaris* isolates from Britain, China, Japan, Korea, and Norway was less than 0.01^10^, lower than the value of 0.04 reported in *Ophiocordyceps sinensis*, an medicinally important fungus that parasitize on ghost moth insects endemic to the Tibetan Plateau^11^. Low genetic variations of the fungus were also observed in mating-type genes *MAT1-1-1* and *MAT1-2-1*^12^. Considering the vast host ranges and its worldwide distribution under various environmental conditions^1^, we expect a genetic diversity at least higher than what’s currently known based on nrDNA ITS. The limited variation of *C. militaris* on nrDNA ITS suggests that the nrDNA ITS region is not an appropriate marker for intraspecific genetic diversity study of *C. militaris* though proved useful in *O. sinensis*^11^. Population and diversity analyses of *C. militaris* require robust and reliable molecular markers.

Compared to nuclear DNA, mitochondrial DNA is much susceptible to damage and mutations mainly because of the presence of reactive oxygen species generated during ATP synthesis^13^. The high mutation number and the faster evolutionary rate of mitochondrial DNA, from 5 to 10 times higher than nuclear DNA^14^, make mitochondrial DNA suitable for discrimination of closely related organisms and elucidation of recent evolutionary events. To date, intraspecific comparative analyses of mitochondrial genome have been performed in some fungal species, including *Candida albicans*^15^, *Lachancea kluyveri*^16^, *Lachancea thermotolerans*^17^, *Mycosphaerella graminicola*^18^, *Neurospora crassa*^19^, *Podospora anserina*^20^, and *Rhizophagus irregularis*^21^. High variability of mitochondrial sequences and sizes were often observed in these fungal species.

Recently, the mitochondrial genome of *C. militaris* was reported^22^, and mitochondrial intron presence/absence dynamics (two to eight introns per strain) was documented for the fungus^23^, indicating a high variability of mitochondrial DNA in the fungus. The objectives for this work were 1) to investigate if there are unknown insertion sites for mitochondrial introns in *C. militaris*, 2) to compare nucleotide variations among different mitochondrial regions, 3) to identify mitochondrial regions with high variability and determine novel molecular markers suitable for typing studies. Therefore, we assembled the complete mitochondrial genomes of eight additional *C. militaris* strains and performed a comparative mitochondrial genomic analysis together with three previously published mitochondrial genomes of the fungus.

## Results

### Overview of different *C. militaris* mitochondrial genomes

**Figure 1:**
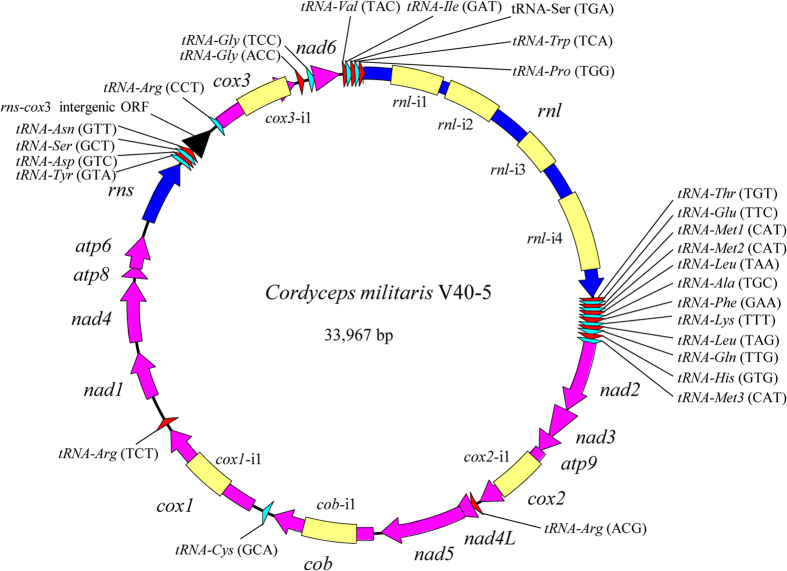
The circular map of the mitochondrial genome of *C. militaris* V40-5. rRNA genes were shown in blue; the 14 standard protein-encoding genes were shown in dark violet; tRNA genes were shown in red or in cyan. Introns were shown in yellow. The *rns*-*cox3* intergenic ORF was shown in black.

The mitochondrial genomes of eight additional *C. militaris* strains were successfully assembled in this study. Together with three strains, CM01, V26–17, and EFCC-C2, whose mitochondrial genomes were reported previously^22^,^23^, we performed a comparative mitochondrial genomic analysis. Each mitochondrial genome consists of two ribosomal RNA genes (*rnl* and *rns*), 27 tRNA genes, and 14 standard protein-coding genes of the oxidative phosphorylation system. Sizes of these mitochondrial genomes, however, varied from 26,535 to 33,967 bp mainly due to different numbers of introns, two to eight depending on strains (Table 1). Compared to our previous publication^23^, no novel insertion sites of introns were found. That is, mitochondrial introns of *C. militaris* seemed to occur only at eight possible sites, four in the *rnl* gene and one each in *cob, cox1, cox2*, and *cox3* (Fig. 1). The second *rnl* intron (i.e., *rnl*-i2) was present in all strains, while each of the remaining seven introns could be absent in one or many strains (Table 1). Every intron contains an ORF encoding for either GIY-YIG homing endonucleases (for *cob*-i1, *cox2*-i1, *rnl*-i1, and *rnl*-i2), the LAGLIDADG homing endonucleases (for *cox1*-i1, *cox3*-i1, and *rnl*-i3), or ribosomal protein S3 (for *rnl*-i4). Besides introns, the *rns*-*cox3* intergenic region (IR) also contributed to mitochondrial genome size variations. This region was 650–661 bp in two strains (EFCC-C2 and CM06) but 1,343 bp in other nine strains. A 327-bp ORF coding for a hypothetical protein was present in the longer *rns*-*cox3* IR, but absent in the shorter *rns*-*cox3* IR. Finally, although trivial, various small insertion/deletion (indel) regions, as depicted in the following sections, also contributed to variations of mitochondrial genome sizes.

### Nucleotide variations at genic regions

Alignment of the 11 *C. militaris* mitochondrial genomes generated 34,067 positions, where approximately 87% comprises genic regions. Nucleotide variations at genic regions were inspected according to exonic (58% of the alignment) and intronic (29%) regions. At exonic regions of the 14 standard protein-coding genes, no indels were detected, but SNPs, from one in *nad3* to 24 in *nad5*, occurred at all genes with exception of *atp8, atp9*, and *nad6* (Table 2). Most SNPs are synonymous, but some from four genes (*cox3, nad2, nad4*, and *nad5*) resulted in one to five amino acid changes. For tRNA genes, only one SNP (either unique to one strain or shared by more than one strain) was detected in each of seven tRNA genes (Table 2); no any variation was found in other tRNA genes. For the two rRNA genes, both SNPs and indels were detected (Table 2). Actually, at exonic regions of all mitochondrial genes, indels were detected just from *rns* and *rnl*, and each indel was unique to one strain.

At intronic regions, no variation was found in *cox3*-i1, but SNPs and often indels were found in the remaining seven introns (Table 3). SNPs from introns of protein-coding genes occurred mostly at intronic ORF regions, while SNPs from introns of *rnl* occurred mostly at regions beyond intronic ORFs. Changes of amino acids were detected for all intronic proteins with exception of those present in *cox3*-i1 and *rnl*-i3, each of which was only possessed by two strains. Indels were found mainly at regions beyond intronic ORFs, but there were two exceptions. One is a 6-bp deletion found in the intronic ORF of *rnl*-i2 from CM09-9-24, which led to the deletion of two amino acids. The other is a truncated version of the intronic ORF of *cox2*-i1 from EFCC-C2, where a 62-bp sequence repeated twice in tandem and led to early translation stop. Unfortunately, mitochondrial sequences of EFCC-C2 reported by Sung (2015) could not be verified in this study.

When we compared the divergence of exons and introns, exons were more divergent than introns in protein-encoding genes, while introns were more divergent than exons in *rnl*. When looking at all intron-containing genes, variability at intronic regions was similar to that at exonic regions (Tables 2 and 3).

### Nucleotide variations at intergenic regions

Intergenic regions only accounted for approximately 13% (5,713–6,415 bp) of *C. militaris* mitochondrial genome, but both SNPs and indels were frequently detected (Table 4). Some indels (e.g., those found at *nad3*-*atp9, cox1*-*nad1*, and *cox3*-*nad6* IRs) were shared by more than one strain. For the *rns*-*cox3* IR, 9 of the 11 strains possessed a large region with an additional ORF, and two strains possessed a short region lacking the ORF. Nucleotide variability at the short *rns*-*cox3* IR, however, was higher than that at the large *rns*-*cox3* IR. When comparing the nucleotide variation frequencies, we found that intergenic regions were more variable than genic regions (Tables 2, 3, 4, [Table t4]).

### Recombination among different mitochondrial regions

The overall index of association for exonic, intronic, and intergenic loci all rejected panmictic recombination (Table S1). Few phylogenetically incompatible locus pairs, however, were found because the proportion of compatible locus pairs was lower than 100%. Manual inspections found allelic combinations showing evidence of recombination between *rnl*-E1 and *rnl*-E2/*nad3/cox2*-E1, between *rnl*-E2 and *nad3/cox2*-E1/*nad4L/nad3*-*atp9* IR/*atp9*-*cox2* IR/*cox2*-*nad4L* IR/*nad5*-*cob* IR (Table S2).

### Development of potential molecular markers

**Figure 2:**
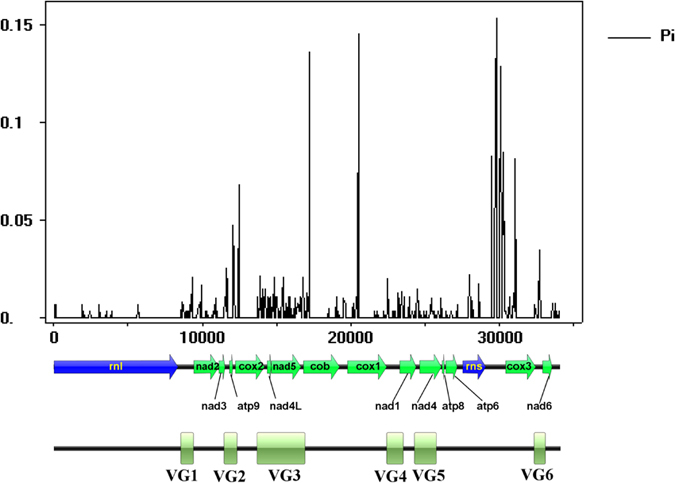
Nucleotide diversity fluctuations across the alignment of 11 *C. militaris* mitochondrial genomes. Gaps were considered during window sliding, but nucleotide variations at gap positions were not considered. The high nucleotide diversity at *rns*-*cox3* intergenic region was due to ambiguous alignment as a result of presence of two different-length sequences among these genomes. The *rns*-*cox3* intergenic region was not considered when selecting novel molecular markers. The relative positions of 14 standard protein-encoding genes, two ribosomal genes, and the six molecular markers were illustrated below the graph.

**Figure 3:**
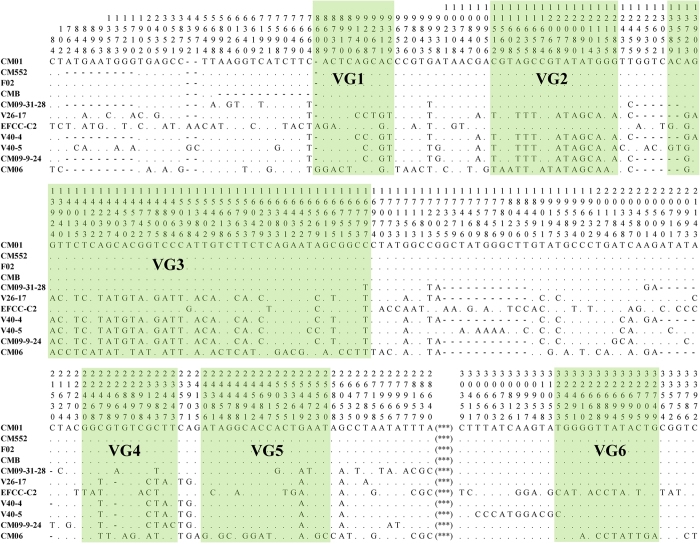
Variable sites across the alignment of 11 *C. militaris* mitochondrial genomes. Digits above nucleotides indicate the nucleotide positions of each polymorphic site in the aligned sequences, reading from top to bottom. Dots mean identical nucleotides to the first sequence; dashes indicate indel. “***” represents an 864-bp sub-region from the *rns*-*cox3* intergenic region; this sub-region was deleted due to ambiguous alignment. A total of 240 variable sites were detected with those present in indels considered. Relative positions of the six variable regions (VG1-6) were indicated in green boxes.

Nucleotide diversity across the alignment of 11 *C. militaris* mitochondrial genomes revealed some mutation hot regions (Figs 2 and 3), such as the *rnl*-*nad2* intergenic region (named VG1), the region from 3′ *nad3* to 5′ *cox2* (VG2), the region from 3′ *cox2* to 5′ *cob* (VG3), the region from 3′ *cox1* to 5′ *nad1* (VG4), the region from 3′ *nad1* to 3′ *nad4* (VG5), and the region from 3′ *cox3* to 5′ *nad6* (VG6). These fragments contained almost half of the total SNP sites detected among the 11 mitochondrial genomes (Fig. 3), showing the potential as novel molecular markers (Table 5).

To determine whether the above six VGs were not under positive selection, we performed the Tajima’s D, and Fu and Li’s D* and F* tests of neutrality for each of them. The values obtained were not significantly different from zero with exception of VG5 (Table 5). Therefore, VGs1–4 and VG6 showed no deviation from the neutral model of evolution and are probably not under selective pressure. Mutations in VG5 are probably affected by natural selection, weakening its potential use in typing studies due to its constrained variability.

**Figure 4:**
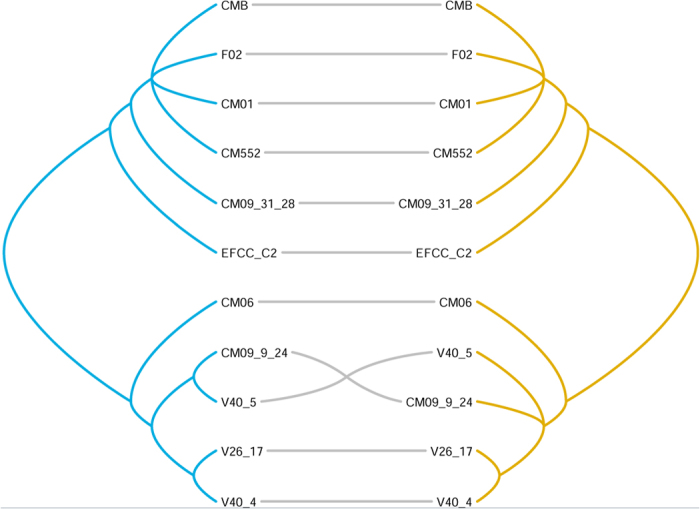
Tanglegram between phylogeny constructed using the whole mitochondrial genome (left) and that constructed using five loci (VGs 1-4, 6; right). Please refer to Table 1 for the information of the 11 *C. militaris* strains used in this figure.

The combined sequence of VGs1–4 and 6 revealed eight haplotypes among the 11 strains (Table 5). When considering the complete mitochondrial genomes, the 11 strains also belonged to eight genotypes. Phylogeny constructed using the concatenated five-locus dataset was congruent to that constructed using the whole mitochondrial genome (Fig. 4). Using designed primers (Table S3), expected bands of the five fragments can be amplified from *C. militaris* strains (Fig. S1).

## Discussion

In this study, we assembled successfully the complete mitochondrial genomes of eight *C. militaris* strains and performed a comparative mitochondrial genomic analysis together with three previously published mitochondrial genomes. All mitochondrial genomes were assembled based on Sanger sequencing results, and sequence errors were reduced to a minimum level as a result of the use of high-fidelity DNA polymerase and the manual check of sequencing chromatograms. The genome alignment of 34,067 positions revealed 286 polymorphic sites in genic regions (222 at exonic regions and >64 at intronic regions) and 160 polymorphic sites in intergenic regions among the 11 *C. militaris* strains (Tables 2, 3, 4, [Table t4]). However, nucleotide variability at intergenic regions was higher than that at genic regions because intergenic regions (~13% of the whole mitochondrial genome) were much shorter than genic regions (~87%). With exception of one to five non-synonymous changes in *cox3, nad2, nad4*, and *nad5*, the majority of mutations in exonic regions of the 14 standard protein-coding genes were synonymous and located in the third codon positions (Table 2). In contrast, intronic ORFs were prone to amino acid changes (Table 3). Overall, variation levels are similar between intronic and exonic regions.

In addition to *C. militaris*, intraspecific comparisons of whole mitochondrial genomes have so far been performed in seven other fungal species^15^,^16^,^17^,^18^,^19^,^20^,^21^. These studies all revealed within-species variations of mitochondrial genomes. Intron presence/absence dynamics were clearly documented in *Lachancea kluyveri*^16^, *Lachancea thermotolerans*^17^, *Podospora anserina*^20^, and *Rhizophagus irregularis*^21^. Higher nucleotide variability at intergenic regions than at genic regions was also documented in *Candida albicans*^15^ and *Lachancea kluyveri*^16^.

Although we detected recombination events between *rnl* exons and some other coding or non-coding regions (Table S2), the overall evidence for panmictic recombination was insignificant (Table S1). Therefore, recombination seems not to contribute a lot to the above observed nucleotide variability. Because the mechanism for mitochondrial inheritance of *C. militaris* has not been investigated, how mitochondrial inheritance affects mitochondrial variations cannot be determined. However, as a heterothallic fungus^6^, there are chances for the generation of heteroplasmons when two *C. militaris* strains with opposite mating types fuse.

As a fungus with a worldwide distribution and a broad host range, *C. militaris* is expected to display a high intraspecific genetic diversity. Previous studies, however, failed to reveal a high intraspecific genetic differentiation based on nuclear DNA fragments^10^. The population genetic structure of *C. militaris* is far from clear due to lack of efficient molecular markers. By comparing mitochondrial genomes of different *C. militaris* strains, this study identified six mitochondrial DNA fragments (VG1–6) with potential as molecular markers. Among them, VG5 was later excluded due to its deviation from neutrality. The remaining five fragments (VG1–4 and VG6) showed the same number of haplotypes as the whole mitochondrial genome and were phylogenetically congruent to the whole mitochondrial genome. We, however, have to admit that *C. militaris* isolates employed in this study were all from China or Chinese companies, more SNPs might be detected when isolates from other countries were tested. Overall, these mitochondrial DNA fragments determined in this study are suitable to be used as molecular markers in future typing studies in *C. militaris*.

## Materials and Methods

### Fungal cultures

Based on our previous study^23^, eight additional *C. militaris* strains that showed multiple intron distribution patterns (Table 1) were chosen to assemble their complete mitochondrial genomes in this study. These isolates were cultivated at 25 °C for 10 days in potato dextrose agar media with a piece of cellophane paper covering the medium surface. Mycelia were collected and used for extracting genomic DNA using the cetyltrimethylammonium bromide method^24^.

### Assembly of mitochondrial genomes and sequence annotations

Based on the published mitochondrial genome of *C. militaris* CM01^23^, multiple PCR primer pairs were designed to amplify mitochondrial fragments from strains used in this study. PCRs were performed using the DNA polymerase KOD FX (TOYOBO Bio-Technology Co. LTD, Japan), and sequences of amplicons were determined by Sanger sequencing at GENEWIZ Inc. (Suzhou, China). Different mitochondrial fragments of the same strain were assembled together under the aid of overlapping sequences. Annotation of mitochondrial genomes referred to those depicted previously^23^.

### Sequence alignment and nucleotide variation analysis

Mitochondrial genome sequences from 11 *C. militaris* strains, eight from this study plus three from previous publications, were aligned by the online program MAFFT version 7 (http://mafft.cbrc.jp/alignment/server/). Individual exonic, intronic, and intergenic regions were extracted from the alignment using a perl script selectSites.pl written by Naoki Takebayashi (http://raven.iab.alaska.edu/~ntakebay/teaching/programming/perl-scripts/perl-scripts.html). Nucleotide variations, including parsimony informative sites, singleton sites, and indels, were summarized for each extracted region in MEGA 6.06^25^.

### Recombination analysis

To know whether mitochondrial recombination might have contributed to the observed nucleotide variations, two complementary tests were conducted to examine evidence for recombination. In the first test, we examined allelic associations among alleles from different loci using the index of association (*I*_A_). In the second test, we calculated the phylogenetic incompatibility by looking for the proportion of loci with all possible recombinant genotypes. Both tests were performed using MultiLocus^26^ with 1,000 randomizations. Twenty-four exonic loci (five *rnl* exonic regions, two *cob* exonic regions, two *cox1* exonic regions, two *cox2* exonic regions, two *cox3* exonic regions, *atp6, atp8, atp9, nad1, nad2, nad3, nad4, nad4L, nad5, nad6*, and *rns*), 8 intronic loci (*rnl*-i1, *rnl*-i2, *rnl*-i3, *rnl*-i4, *cob*-i1, *cox1*-i1, *cox2*-i1, and *cox3*-i1), and 15 intergenic loci (i.e., those among 14 standard protein-coding genes and ribosomal RNA genes) were examined.

### Development of molecular markers

Nucleotide diversity across the alignment of 11 mitochondrial genomes was calculated in DnaSP 5.10^27^ using a sliding window of 50-bp window length and 50-bp step size with alignment gaps counted in the window length and slide. Regions with dense distribution of single nucleotide polymorphism (SNP) sites were investigated as potential molecular markers.

### Neutrality test

For the above-selected markers, statistical tests of Tajima’s D and Fu and Li’s D* and F* for detection of deviation from the neutral model of evolution were performed using DnaSP 5.10^27^. Those without significant deviation from neutrality are suitable as molecular markers in future typing studies.

### Phylogenetic tree construction and comparison

To know if the novel molecular markers are phylogenetically congruent to the complete mitochondrial genomes, we constructed phylogenetic trees using two datasets. One is the combined dataset of new markers chosen in the above section, and the other is the complete mitochondrial genomes but with partial *rns*-*cox3* intergenic region excluded because of ambiguous alignment. Maximum parsimony trees were constructed using PAUP 4.0b10 (Sinauer Associates, Sunderland, MA, USA) with gaps being treated as missing data. A tanglegram was constructed from both trees using TREEMAP 3b1243^28^.

### Nucleotide accession numbers

Sequences of the eight newly assembled mitochondrial genomes of *C. militaris* were all submitted to GenBank under accession numbers as depicted in Table 1.

## Additional Information

**How to cite this article:** Zhang, S. *et al*. Comparative mitochondrial genomics toward exploring molecular markers in the medicinal fungus *Cordyceps militaris. Sci. Rep.* **7**, 40219; doi: 10.1038/srep40219 (2017).

**Publisher's note:** Springer Nature remains neutral with regard to jurisdictional claims in published maps and institutional affiliations.

## Supplementary Material

Supplementary Information

## Figures and Tables

**Table 1:** *C. militaris* isolates used in this study and information on their mitochondrial genomes.

Isolate	Source	GenBank accession no.	Size (bp)	Intron^a^								No. introns	*rns-cox3* IR^a^	Clade^b^
				*cob*-i1	*cox1-*i1	*cox2-*i1	*cox3-*i1	*rnl-*i1	*rnl-*i2	*rnl-*i3	*rnl-*i4			
V26-17	Company	KP719097	29478		+			+	+		+	4	+	A
V40-4	Company	KP722512	27235						+		+	2	+	B
V40-5	Company	KP722501	33967	+	+	+	+	+	+	+	+	8	+	D
CM09-9-24	Liaoning, China	KP722496	28285		+				+		+	3	+	C
CM09-31-28	Liaoning, China	KP722513	27245						+		+	2	+	B
CM552	Liaoning, China	KP722502	30662	+	+	+			+		+	5	+	E
CM01	unknown	KP719096	31854	+	+	+		+	+		+	6	+	F
CM06	unknown	KP722500	26535						+		+	2		B
F02	unknown	KP722505	31854	+	+	+		+	+		+	6	+	F
CMB	unknown	KP722507	30026	+	+	+		+	+			5	+	G
EFCC-C2	unknown	NC_022834	33277	+	+	+	+	+	+	+	+	8		D

^a^“+” Indicates the presence of an intron or the large *rns*-*cox3* intergenic region (IR).

^b^The clade here referred to those classified according to intron presence/absence patterns as depicted in our previous paper (Zhang *et al*. 2015).

**Table 2:** Variations at exonic regions of each mitochondrial gene among 11 *C. militaris* strains.

Gene	Exon						Protein				
	Length (bp)	Pi	S	Indel	Total	%	Length (aa)	Pi	S	Indel	Total
*atp6*	783	1	3	0	4	0.5	260	0	0	0	0
*atp8*	147	0	0	0	0	0.0	48	0	0	0	0
*atp9*	225	0	0	0	0	0.0	74	0	0	0	0
*cob*	1161	6	6	0	12	1.0	386	0	0	0	0
*cox1*	1593	4	7	0	11	0.7	530	0	0	0	0
*cox2*	750	7	2	0	9	1.2	249	0	0	0	0
*cox3*	810	5	7	0	12	1.5	269	1	0	0	1
*nad1*	1104	3	4	0	7	0.6	367	0	0	0	0
*nad2*	1686	4	8	0	12	0.7	561	1	4	0	5
*nad3*	420	1	0	0	1	0.2	139	0	0	0	0
*nad4*	1449	2	9	0	11	0.8	482	2	1	0	3
*nad4L*	270	3	0	0	3	1.1	89	0	0	0	0
*nad5*	1995	12	12	0	24	1.2	664	1	1	0	2
*nad6*	633	0	0	0	0	0.0	210	0	0	0	0
*rns*	1538–1558	3	1	23	27	1.7					
*rnl*	3120–3189	4	3	75	82	2.6					
*tRNA-Leu* (TAA)	82	1	0	0	1	1.2					
*tRNA-Phe* (GAA)	73	0	1	0	1	1.4					
*tRNA-Lys* (TTT)	73	0	1	0	1	1.4					
*tRNA-Gln* (TTG)	73	1	0	0	1	1.4					
*tRNA-His* (GTG)	73	0	1	0	1	1.4					
*tRNA-Arg* (ACG)	71	1	0	0	1	1.4					
*tRNA-Arg* (CCT)	71	0	1	0	1	1.4					
Other 20 tRNAs	1481	0	0	0	0	0.0					
Total	19681–19770	58	66	98	222	1.1	4328	5	6	0	11

Pi, parsimony informative sites; S, singleton sites; indel, insertion/deletion sites; %, the percentage of total variable sites (Pi, S, and indels) in each gene.

**Table 3:** Variations at intronic regions of mitochondrial genes among 11* C. militaris* strains.

Intron	No. strains	Intron						Intronic ORF						Intronic protein				
		Length (bp)	Pi	S	Indel	Total	%	Length (bp)	Pi	S	Indel	Total	%	Length (aa)	Pi	S	Indel	Total
*cob-*i1	6	1256–1257	2	9	1	12	1.0	918	1	8	0	9	1.0	305	0	5	0	5
*cox1*-i1	8	1051	2	4	0	6	0.6	945	2	3	0	5	0.5	314	0	1	0	1
*cox2-*i1^a^	6	1125–1187	1	5	62 (0)	68 (6)	5.7 (0.5)	861	0	4	0	4	0.5	286	0	3	0	3
*cox3-*i1	2	1213	0	0	0	0	0.0	933	0	0	0	0	0.0	310	0	0	0	0
*rnl-*i1	6	1191–1192	2	7	1	10	0.8	738	1	3	0	4	0.5	245	1	1	0	2
*rnl-*i2	11	1235–1241	1	2	6	9	0.7	756–762	1	1	6	8	1.0	251–253	1	1	2	4
*rnl*-i3	2	893	0	2	0	2	0.2	624	0	1	0	1	0.2	207	0	0	0	0
*rnl*-i4	10	1821–1828	1	11	7	19	1.0	1323	0	4	0	4	0.3	440	0	2	0	2
Total		3062–9861	9	40	77 (15)	126 (64)		7098–7104	5	24	6	35	0.5	2358–2360	2	13	2	17

^a^The large number of indels present in *cox2*-i1 was due to an extra tandem repeat of a 62-bp fragment (at 200 bp 5′ normal stop codon), which was present merely in the strain EFCC-C2 and led to early translation stop of the intronic ORF. We could not confirm sequences from this strain, therefore, variations in *cox2*-i1 were also calculated by deleting this extra repeat and values after the repeat deletion were given within parenthesis. This repeat was also represented only once when calculating nucleotide variation indices for intronic ORF and intronic protein in this table.

**Table 4 t4:** Variations at intergenic regions of mitochondrial genes among 11* C. militaris* strains.

Intergenic region^a^	No. strains	Actual length (bp)	Remaining length (bp)^c^	Pi	S	Indel	Total	%^c^	Note
*rnl-nad2*	11	1056–1065	166–175	4	0	9	13	7.6	12 tRNAs excluded
*nad3-atp9*	11	278–286	278–286	3	3	8	14	4.9	
*atp9-cox2*	11	176–186	176–186	7	2	10	19	10.5	
*cox2-nad4L*	11	200–201	129–130	1	1	1	3	2.3	1 tRNA excluded
*nad5-cob*	11	183	183	2	3	0	5	2.7	
*cob-cox1*	11	529–536	457–464	1	4	9	14	3.0	1 tRNA excluded
*cox1-nad1*	11	866–879	795–808	3	6	21	30	3.7	1 tRNA excluded
*nad1-nad4*	11	242	242	0	5	0	5	2.1	
*nad4-atp8*	11	67	67	0	0	0	0	0.0	
*atp8-atp6*	11	80	80	0	0	0	0	0.0	
*atp6-rns*	11	356–358	356–358	0	0	3	3	0.8	
large *rns-cox3* IR^b^	9	1343	963	0	1	0	1	0.1	5 tRNAs excluded
small *rns-cox3* IR^b^	2	650–661	270–281	0	5	11	16	5.8	5 tRNAs excluded
*cox3-nad6*	11	436–457	293–314	5	6	21	32	10.5	2 tRNAs excluded
*nad6-rnl*	11	551	178	0	5	0	5	2.8	5 tRNAs excluded
Total		5713–6415	3713–4415	26	41	93	160	3.6–4.3	27 tRNAs excluded

^a^Intergenic regions are named by adjacent protein-encoding genes and/or ribosomal genes. tRNAs present in some of these fragments were excluded during calculation as noted in this table.

^b^For the large *rns*-*cox3* IR and the small *rns*-*cox3* IR, identical five tRNAs were excluded. Therefore, the total number of tRNAs was still 27.

^c^“Remaining length” represents the length after excluding tRNAs. % was calculated based on remaining length.

**Table 5:** Nucleotide variations and neutrality tests on the six fragments chosen as potential molecular markers.

Locus	Actual length (bp)	Aligned length (bp)	Pi	S	Indel	Total	%	No. alleles	Nucleotide diversity^a^	Haplotype diversity ^a^	Tajima’s D	Fu & Li’s D*	Fu & Li’s F*	*P* value^b^
VG1	875–884	884	6	3	9	18	2.04	6	0.0035 ± 0.0007	0.800 ± 0.114	0.0248	0.1597	0.1420	>0.1
VG2	878–888	888	11	5	18	34	3.83	3	0.0079 ± 0.0013	0.618 ± 0.104	1.1208	0.2556	0.5389	>0.1
VG3	3230–3231	3231	25	17	1	43	1.33	7	0.0050 ± 0.0008	0.873 ± 0.089	0.6392	−0.1260	0.0798	>0.1
VG3A	1509–1510	1510	16	3	1	20	1.32	5	0.0059 ± 0.0008	0.782 ± 0.107				
VG3B	1850	1850	9	14	0	23	1.24	6	0.0039 ± 0.0009	0.836 ± 0.089				
VG4	1119–1132	1132	4	7	21	32	2.83	6	0.0031 ± 0.0005	0.836 ± 0.089	−0.6617	−1.1442	−1.1561	>0.1
VG5	1474	1474	1	15	0	16	1.09	5	0.0022 ± 0.0009	0.782 ± 0.093	−1.8166	−2.2777	−2.4466	<0.05
VG6	750–771	771	6	7	21	34	4.41	4	0.0043 ± 0.0020	0.491 ± 0.175	−1.1854	−0.6463	−0.8864	>0.1
Combined 6-locus dataset	8348–8365	8380	53	54	70	177	2.11	8	0.0044 ± 0.0008	0.891 ± 0.092				
Combined 5-locus dataset excluding VG5	6874–6891	6906	52	39	70	161	2.33	8	0.0048 ± 0.0008	0.891 ± 0.092				

^a^Numbers after “±” were standard deviations.

^b^*P* values are those for denying neutrality.
