# Supplementary material for: Comparative mitochondrial genomics toward exploring molecular markers in the medicinal fungus Cordyceps militaris
Source: Sci Rep. 2017 Jan 10;7:40219. doi: 10.1038/srep40219 (PMC5223169; doi:10.1038/srep40219)
Supplement: Supplementary Information [file srep40219-s1.pdf]

Comparative mitochondrial genomics toward exploring molecular markers in the medicinal fungus *Cordyceps militaris*

Shu Zhang [1, 2], Ai-Jing Hao [2], Yu-Xiang Zhao [2], Xiao-Yu Zhang [3], Yong-Jie Zhang [2 *]

[1] Institute of Applied Chemistry, Shanxi University, Taiyuan 030006, China;

[2] School of Life Sciences, Shanxi University, Taiyuan Shanxi 030006, China

[3] Farm Product Storage and Freshening Institute, Shanxi Academy of Agricultural Sciences, Taiyuan Shanxi 030031, China

Correspondence and requests for materials should be addressed to Y.J.Z. (email: zhangyj2008@sxu.edu.cn)

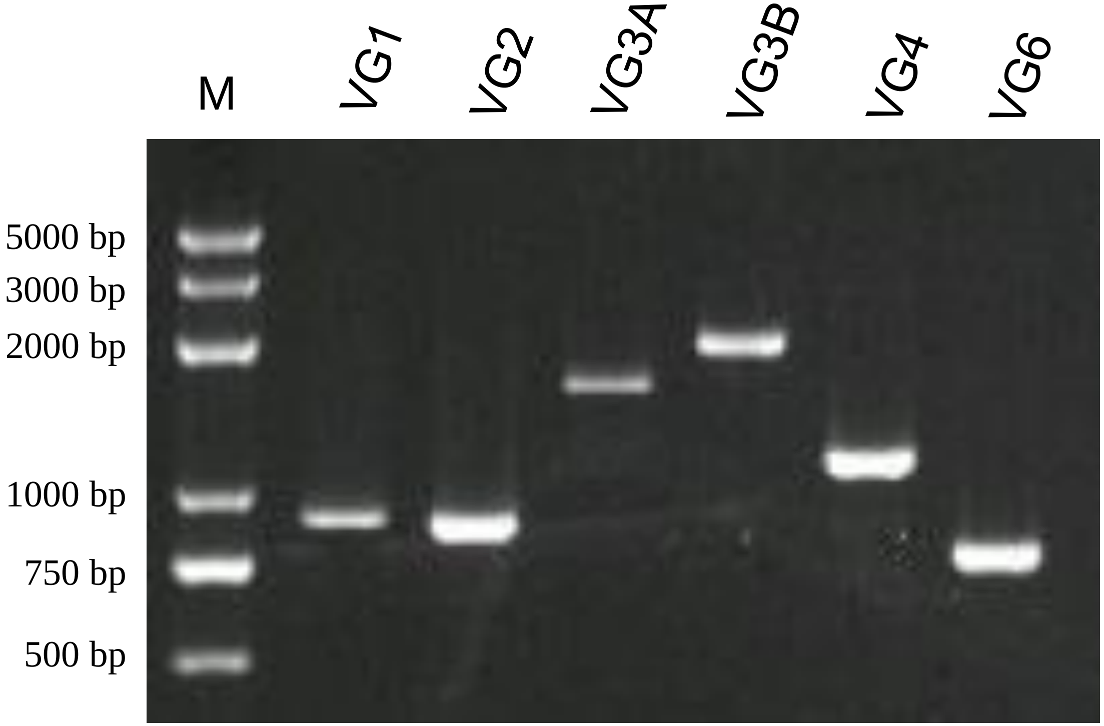

Fig. S1 Electrophoretic pattern of five molecular markers (VG1-4, 6) from a representative *C. militaris* strain. The fragment VG3 was amplified by dividing it into two small fragments VG3A and VG3B due to its length. Amplifications in this figure were all performed with the *C. militaris* strain CM06 as templates.

Table S1. Recombination analysis of various regions

	No. loci	PrCP	Index of association	*P* value
Exonic loci	24	0.978	6.444	< 0.001
Intronic loci	8	1.000	1.595	< 0.001
Intergenic loci	15	1.000	4.306	< 0.001
Exonic & intronic loci	32	0.988	7.693	< 0.001
Exonic & intergenic loci	39	0.987	11.115	< 0.001
Intronic & intergenic loci	23	1.000	4.919	< 0.001
Exonic & intronic & intergenic loci	47	0.991	11.975	< 0.001

PrCP, percentage of compatible locus pairs; IA, index of association. P values are those to reject the null hypothesis of random recombination.

Table S2. Loci showing evidence of recombination

Strain	*rnl* -E1	*rnl* -E2	*nad3*	*cox2* _E1	*nad4L*	*nad3-atp9* IR	*atp9-cox2* IR	*cox2-nad4L* IR	*nad5-cob* IR
V26-17	1	2	2	2	2	2	2	2	2
V40-4	1	1	2	2	2	2	2	2	2
V40-5	1	1	2	3	2	2	2	3	2
CM09-9-24	1	1	2	2	2	2	2	2	2
CM09-31-28	1	1	1	2	1	3	1	1	3
CM552	1	1	1	1	1	1	1	1	1
CM01	1	1	1	1	1	1	1	1	1
CM06	2	1	2	2	3	4	3	4	4
F02	1	1	1	1	1	1	1	1	1
CMB	1	1	1	1	1	1	1	1	1
EFCC-C2	2	2	1	1	1	3	1	1	3

Notes: Numbers in this table are allele identifiers. IR, intergenic region

rnl -E1 showed recombination with other loci shown in yellow.

rnl -E2 showed recombination with other loci shown in blue.

Table S3. Primers designed to amplify marker fragments

Locus	Primer name	Primer sequence	Direction	Size (bp)	Notes
VG1	VG1-F	GTTCGATCCCTGTCTAGTCTAT	F		
	VG1-R	TGAGATCAGTGTTCTAACCGTT	R	884	within *rnl* -*nad2* IR
VG2	VG2-F	TATTCGAATTAGGTAAAGGTGCT	F		
	VG2-R	TGGAGTAGCACTATCTTGGAAAT	R	888	3' *nad3* --5' *cox2*
VG3A	VG3A-F	ATATGAACTATTACACCGGCAT	F		
	VG3A-R	AAGAAAGCATCACCAACTCTAT	R	1510	3' *cox2* -- 5' *nad5*
VG3B	VG3B-F	AATGTTTGTAGGATGAGAAGGT	F		
	VG3B-R	TATTAGTTGGTTGTGAATGATCA	R	1850	5'-*nad5* -- 5' *cob*
VG4	VG4-F	CTAAACCTCATGCATTCGTAAG	F		
	VG4-R	GCTTGAGTAGGTGCTATATATTC	R	1132	3’ *cox1* to 5’ *nad1*
VG6	VG6-F	GTACAATATTCTTAGCAGTAGGT	F		
	VG6-R	TGAAATCGAAGCAAATAATCCT	R	771	3’ *cox3* to 5’ *nad6*

The fragment VG3 was amplified by combining two small fragments VG3A and VG3B.
